# Absorption of Nutrient Enemata

**Published:** 1883-03

**Authors:** 


THE ABSORPTION OF NUTRIENT ENEMATA.—Dr. Charles L. Dana, of New York, communicates to the *Medical Record* the results of a series of experiments upon dogs, made to determine the physiological process by which nutrient enemata are absorbed. He finds that large injections forcibly administered *may* cause a "retrostasis," which will carry the material by the ileo-cæcal valve, and even into the stomach; moreover, that, in dogs, ordinary nutrient injections of two, three, or four ounces pass back some distance, and may even reach the ileo-cæcal valve, but do not go farther, the injection being carried back much better when the lower bowel is empty or comparatively so. He believes that the clinical cases are exceptional in which retroperistalsis occurs, and concludes that the process is usually effected by local absorption. The fact that the colon is very vascular and has a large supply of lymphatics confirms the view that it is not an excreting organ, but its function in man is that of absorbing: in solipeds it has a powerful digestive action. He observes that albuminous food, when injected, speedily undergoes chemical changes and decomposition. In some of the early stages of this process it is quite possible that the changed albumen passes into the surrounding vessels. Normal peptic digestion is only a decomposition with many stages in it, during some of which the albuminous matter is absorbed. It is not necessary that albumens be made perfect peptones before they can diffuse into the blood vessels and lymphatics. Fats cannot be absorbed to any great extent in the colon or rectum. It is not necessary to inquire whether starches can be changed to glucose, since it is always possible to add some form of animal sugar to the enema if that be thought necessary. Milk and beef tea are regarded as very nearly as effective as the expensive peptonized preparations for use in nutrient enemata, the value of which may be regarded as established both by physiological experiment and abundant clinical experience.—*Medical Times.*

